# Prevalence, risk factors and first record of mitochondrial *cox*1 gene-based molecular characterization of *Paramphistomum epiclitum* from Pakistan

**DOI:** 10.3389/fvets.2022.1018854

**Published:** 2022-11-21

**Authors:** Mughees Aizaz Alvi, Ayed Alshammari, Faizan Asghar, Rana Muhammad Athar Ali, Li Li, Muhammad Saqib, Muhammad Kasib Khan, Muhammad Imran, Warda Qamar, Hussam Askar, Naser Abdelsater, Bao-Quan Fu, Hong-Bin Yan, Wan-Zhong Jia

**Affiliations:** ^1^Key Laboratory of Veterinary Etiological Biology, College of Veterinary Medicine, Lanzhou University, National Para-Reference Laboratory for Animal Echinococcosis, Lanzhou Veterinary Research Institute, Chinese Academy of Agricultural Sciences, Lanzhou, China; ^2^Department of Clinical Medicine and Surgery, University of Agriculture, Faisalabad, Pakistan; ^3^Department of Biology, College of Science, University of Hafr Al Batin, Hafr Al Batin, Saudi Arabia; ^4^Department of Parasitology, University of Agriculture, Faisalabad, Pakistan; ^5^Faculty of Science, Al-Azhar University, Assuit, Egypt; ^6^Jiangsu Co-innovation Center for Prevention and Control of Important Animal Infectious Diseases and Zoonoses, Yangzhou, China

**Keywords:** prevalence, risk factors, genetic diversity, *cox*1, sheep and goat, Pakistan

## Abstract

Parasitic infestations are one of the major threats to the livestock industry in Pakistan. These have a negative impact on the production of domesticated livestock species. Paramphistomes belong to the superfamily Paramphistomoidea and are involved in infecting ruminants all over the world. To date, there was no information on mitochondrial DNA-based molecular characterization of *Paramphistomum epiclitum* from Pakistan. To close this research gap, this study was designed to provide insights into the epidemiology of *Paramphistomum* species. *Paramphistomum epiclitum* isolates were recovered from the rumen of small ruminants slaughtered at an abattoir located in Faisalabad city and animal demographics were recorded. DNA was extracted and mitochondrial *cox*1 was amplified and sequenced. Prevalence was calculated along with a 95% confidence interval in various groups. The chi-square test was applied to determine the association between different variables under investigation. A phylogenetic tree was constructed based on the Bayesian method. Population diversity indices were calculated using DnaSP 4.5 software. A total of 43 mutations were observed among 7 haplotypes. Negative values of Fu's Fs values, and Tajima's D indicated population expansion. Deworming, season, and grazing were the variables that significantly correlate (*p* < 0.05) with the prevalence of *P*. *epiclitum*. The high prevalence of *P. epiclitum* demonstrates that more studies are indeed needed to further understand the prevalence and distribution of *P. epiclitum* in definitive and all potential intermediate hosts in addition to intraspecies variation and relationship with populations from other locations.

## Introduction

*Paramphistomum* infection is one of the neglected parasitic diseases affecting ruminants, which is widely distributed in tropical and subtropical areas of the world. *Paramphistomum* is derived from the Greek word amphistomes, which means paired mouth (1). The life cycle of *Paramphistomum* is completed between a snail (*Planorbis planorbis, Bulinus spp*., and *Lymnaea bulimoides*) as intermediate host and ruminants as definitive host (2). Miracdia are released from the eggs shed in the feces of infected animals. These miracidia invade snails. Parasitic larvae are developed in snails until cercaria is produced and these cercariae then encyst on hard surfaces, vegetation, or in water. Infective metacercariae are ingested by ruminants. Immature larvae present in the small intestine move to their predilection site such as fore stomach and liver where they are converted into adult flukes (3–5).

Parasites are capable of causing acute, chronic, and debilitating types of diseases leading to production losses in animals and researches have shown the positive role of alternative/complementary medicine to treat parasitic diseases (6–9). Generally, adult paramphistomes are considered non-pathogenic but anorexia, diarrhea, polydipsia, and mortality are caused by hemorrhagic enteritis due to migration of the larvae in duodenal mucosa. Paramphistomosis is caused by several species in different regions of the world but *P. cervi* and *P*. *epiclitum* are considered to be the most important species (10, 11). The prevalence of different *Paramphistomum* species has been reported in various species like sheep, goats, cattle, buffalo, and camels. The prevalence of *Paramphistomum spp*. in cattle was reported to be 36.9% in South-Eastern Iran (12). *Raza et al*. reported the prevalence of *P. cervi* to be 28.57, 23.80, 17.64, and 20% in sheep, goat, cattle, and buffalo, respectively (13). Prevalence of *P. cervi* was found to be 34% in camels reported from Maiduguri, Nigeria (14).

*Paramphistomum epiclitum* is the plug feeders (15) that burry themselves in the duodenal mucosa and feed on cells of Brunner's gland leading to diarrhea, anemia, hypoproteinemia, and weakness (16). The infection results in a poor feed conversion ratio, poor weight gain, and decreased milk production in affected animals (17). Recently the parasite has been found to cause significant losses in production (10, 18). Prevalence is high in tropical and subtropical areas, especially in Asia, Africa, and Eastern Europe (13, 19, 20). The prevalence in some areas of Asia such as Pakistan is recorded to be 30–60% (13). The prevalence of gastrointestinal helminths such as *P. epiclitum* in Pakistan has been previously recorded to be 25.1–92% in different regions at different times (13, 21–23).

Pakistan is endowed with a rich population of small ruminants that account for more than 100 million heads (24). One of the threatening issues faced by the farmers is a parasitic infection which results in decreased productivity of animals in terms of losses in weight gain, milk production, and reproduction of the infected animals. While addressing any problem one has to determine the magnitude of that problem to devise suitable and effective prevention and control strategies. Prevalence studies help to determine a true picture of diseases within the population of animals inhabiting that area. Currently, a few studies have been conducted on the prevalence of *P. epiclitum* in different areas of Pakistan but there is a lack of a comprehensive prevalence study on *P. epiclitum* in the Faisalabad region. Previously, numerous reports on molecular characterization based on the ITS gene are available from the country but *the ITS* gene is a hypervariable region and thus does not a candidate gene to molecularly characterize and identify the species. Similarly to date, no information on mitochondrial DNA-based molecular characterization is available from the region. To close these research gaps, the current study was designed.

## Materials and methods

### Study area

Pakistan is a South Asian country that shares its border with Iran in the southwest, China in the northeast, India in the east, and Afghanistan in the west. Faisalabad is the third most populous city in Pakistan and the second most populous city in Punjab Province. Geographically it is expanded from 730 to 740 in the east and 300 to 31.50 in the north over an area of 5,856 km2 and lies 186 meters above sea level. Annual rainfall in Faisalabad is about 350 millimeters. It is known for its well-developed canal system. The existence of a highly extensive irrigation system makes this land highly valuable for agriculture as well as livestock farming. The sampling of *P. epiclitum* was done from a central abattoir located in Faisalabad, Punjab Province. This slaughterhouse is a source of quality meat supply over the major area of the city which is why a huge number of small ruminants are pooled into this abattoir for slaughtering.

### Parasite collection

From February 2021 till the end of January 2022, a total of 1,942 animals were examined at the abattoir post-slaughter. Sampling was done 5 days a week except for Tuesday and Wednesday due to weekly holidays in the slaughterhouse. One parasite per animal was collected from the animals found positive for *P. epiclitum* infection in Eppendorf tubes and data (species *viz*. sheep or goat, sex, age, breed, grazing status, flock size, and deworming status) of all the animals were recorded before slaughtering. Samples were labeled and transported to the Department of Clinical Medicine and Surgery, University of Agriculture, Faisalabad, Pakistan for further processing.

### DNA extraction and PCR

DNA was extracted from a total of 40 *P*. *epiclitum* isolates using Qiagen^®^ Blood and Tissue Kit strictly adhering to the manufacturer's guidelines. The final reaction mixture used for PCR contained a total volume of 50 μl comprising 10 pmol of each primer, 25 μl Premix Ex Taq™ version 2.0, 0.5 μl of sample DNA extract (≥20 ng), and volume made up to 50 μl by adding sufficient amount of DNase/RNase free distilled water (UltraPureTM, Introgen). PCR reaction mixture used as control negatives were added with nuclease-free water instead of DNA. Previously used primers JB4.5 (5′-TAAAGAAAGAACATAATGAAAATG-3′) and JB3 (5′-TTTTTTGGGCATCCTGAGGTTTAT-3′) were used to amplify the *cox*1 gene (25). PCR conditions used were as follows: initial denaturation at 94°C for 5 min followed by 35 cycles of 30 s at 94°C, 45 s at 50°C, and 35 s at 72°C, and a final extension at 72°C for 10 min (26). PCR products were run on 1.5% (*w/v*) agarose gel stained with GelRed™ and the products were viewed under the GelDoc system. A 500 bp ladder was used in each gel as a DNA marker for estimating the size of amplicons. Then PCR products were sent to Beijing Tsingke Biotechnology Co., Ltd., Beijing, China for sequencing.

### Molecular analysis

DNA sequences were viewed and misread nucleotides were corrected as well as nucleotide sequences were aligned using Unipro UGENE v1.32.0 software while the identity and nucleotide sequence of each isolate was confirmed by using NCBI BLAST Program (27). By viewing the electropherogram using Chromas software, the low-quality parts at the beginning and end were trimmed. This was done to discard mismatching extremities. Population diversity indices that included haplotype diversity (Hd), no. of haplotypes (h), and nucleotide diversity (π) were calculated by using DnaSP 4.5 software (28). Neutrality indices of Tajima's D and Fu's Fs were determined using Arlequin 3.5.2.2 software (29–31). Nucleotide sequences of the *cox*1 gene of 7 haplotypes were used to construct a Bayesian phylogenetic tree using MrBayesv.3.1.1 software, taking *Schistosoma japonicum* as outgroup (32).

### Statistical analysis

For analyzing data, chi-square values and Odds ratios were calculated using Statistix 10.0 and WinPepi software, respectively. The *p* < 0.05 was considered significant.

## Results

Higher prevalence was found in sheep (28.6%) as compared to goats (24.50%) but the difference in the prevalence between sheep and goats was statistically non-significant (*p* > 0.05) (Table 1). The highest prevalence was observed in animals with age < 1 year (28.57%) followed by age groups aging >4 years and 1–2 years having a prevalence of 27.51 and 23.71%, respectively. The lowest prevalence (22.85%) was found in animals aging between 2 and 4 years. There was no statistically significant difference (*p* > 0.05) in prevalence among various age groups. Concerning the sex of the animals, males showed a higher prevalence (23.98%) as compared to females (28.73%) and the difference was non-significant (*p* > 0.05).

**Table 1:** Prevalence of *Paramphistomum epiclitum* in small ruminants.

**Variable**	**Category**	**Positive/tested**	**Prevalence (95% CI)**	**Chi–square** **(*P*–value)**	**OR**	**95% CI**
Species	Sheep	135/472	28.6 % (24.61–32.95)	1.86 (0.1729)	1.17	0.93–1.46
	Goat	360/1470	24.50 % (22.33–26.79)		–	–
Age	<1 year	176/616	28.57% (25.16–32.26)	3.77 (0.2890)	1.25	0.96–1.63
	>4 year	63/229	27.51% (22.13–33.63)		1.20	0.85–1.70
	1–2 year	147/620	23.71% (20.53–27.21)		1.04	0.79–1.37
	2–4 year	109/477	22.85% (19.31–26.83)		–	–
Sex	Female	177/616	28.73% (25.3–32.43)	2.93 (0.0870)	1.20	0.97–1.27
	Male	318/1326	23.98% (21.76–26.35)		–	–
Grazing status	Grazing	399/1367	29.19% (26.84–31.66)	21.27 (<0.001)	1.17	1.38–2.25
	Non grazing	95/575	16.52% (13.71–19.78)		–	–
Deworming status	Not dewormed	380/797	47.68% (44.23–51.15)	294.35 (<0.001)	4.75	3.78–5.96
	Dewormed	115/1145	10.04% (8.43–11.92)		–	–
Flock size	>15	285/1074	26.54% (23.99–29.26)	1.17 (0.557)	1.14	0.90–1.45
	10–15	95/373	25.47% (21.31–30.13)		1.10	0.81–1.48
	5–10	115/495	23.23% (19.72–27.15)		–	–
Breed	Buchi	39/120	32.5% (24.78–41.31)	5.97 (0.3086)	1.51	0.97–2.36
	Kajli	75/270	27.78% (22.78–33.41)		1.29	0.90– 1.87
	Cross bred	198/ 729	27.16% (23.99–30.57)		1.26	0.93–1.72
	Lohi	21/82	25.61% (17.4–36.0)		1.19	0.69–2.06
	Teddy	95/429	22.14 % (18.36–26.43)		1.03	0.73–1.46
	Beetal	67/312	21.47% (17.13–26.53)		–	–
Season	Autumn	184/498	36.95% (32.83–41.27)	38.635 (<0.00001)	2.16	1.63–2.86
	Summer	96/310	30.97% (26.08–36.32)		1.81	1.31–2.49
	Spring	127/620	20.48% (17.49–23.83)		1.20	0.89–1.61
	Winter	88/514	17.12% (14.11–20.62)		–	–

The prevalence of *P*. *epiclitum* in grazing and stall-fed animals was 29.19 and 16.52%, respectively. Grazing animals had significantly (*p* < 0.05) higher rates of infection than stall-fed animals. Similarly, animals that were not treated with any anthelmintic showed a statistically significant difference (*p* < 0.05) from those which had a history of deworming medication. Seasonal calculation of prevalence showed a significant (*p* < 0.05) relationship between season and prevalence of *P. epiclitum*. Prevalence was found to be highest during the Autumn months (36.95%; September to November) followed by Summer (30.97%; June to August), and Winter (17.12%; December to February).

While comparing the prevalence of *P. epiclitum* among different breeds of sheep and goats, the prevalence was calculated to be highest in Buchi sheep and least in Beetal goats having a prevalence of 32.5 and 21.47%, respectively. No statistically significant difference in prevalence was found among various breeds of sheep and goat.

Amplification of the *cox*1 gene yielded PCR products of approximately 450 bp. Nucleotide sequences of all 40 isolates analyzed in this study were aligned with reference sequences of *P. epiclitum*, retrieved from GenBank. The newly generated sequences of *P. epiclitum* showed a total of 43 mutation sites. Among the 40 *P. epiclitum* isolates, 7 haplotypes were found for the *cox*1 gene. Table 2A shows the observed nucleotide polymorphism between haplotypes while the resulting amino acid changes are mentioned in Table 2B.

**Table 2:** *Paramphistomum spp*. partial *cox*1 gene nucleotide sequence polymorphism and corresponding amino acid changes.

	**1**	**4**	**7**	**8**	**11**	**16**	**20**	**28**	**31**	**53**	**66**	**88**	**94**	**97**	**10**	**106**	**121**	**142**	**145**	**151**	**157**	**159**	**160**	**163**	**176**	**179**	**181**	**197**	**198**	**205**	**220**	**223**	**224**	**229**	**239**	**244**	**248**	**250**	**253**	**265**	**266**	**271**	**274**
**A**. ***cox*****1 mutation sites**.																																											
UAF1	G	T	T	T	T	T	T	G	G	T	T	C	G	T	A	C	C	C	A	A	A	G	G	A	T	T	A	A	C	C	G	G	A	G	A	T	G	A	G	A	A	G	G
UAF2	.	.	.	.	.	.	.	.	.	.	.	.	.	.	.	.	.	.	.	.	.	A	.	.	.	.	.	.	.	.	.	.	.	.	G	.	.	.	.	.	G	.	.
UAF3	.	A	.	C	C	.	.	.	.	C	C	.	.	.	.	.	.	.	.	.	.	A	.	.	C	.	.	.	.	.	.	.	.	.	G	.	.	.	.	.	G	.	.
UAF4	.	.	.	.	.	.	.	.	.	.	.	.	.	.	.	.	.	.	.	.	.	.	.	.	.	.	.	.	.	.	.	.	.	.	G	.	.	.	.	.	G	.	.
UAF5	.	.	.	.	.	.	A	.	.	.	.	.	.	.	.	.	.	.	.	.	.	A	.	.	.	.	.	.	.	.	.	.	.	.	.	.	.	.	.	.	G	.	.
UAF6	A	.	G	.	.	C	.	T	A	.	.	T	T	G	G	G	T	A	T	T	T	.	A	T	.	C	G	G	T	T	T	T	G	C	G	C	A	T	A	T	G	A	T
UAF7	.	.	.	.	C	.	.	.	.	.	.	.	.	.	.	.	.	.	.	.	.	.	.	.	.	.	.	.	.	.	.	.	.	.	G	.	.	.	.	.	G	.	.
	**1**		**2**		**3**		**4**		**10**		**11**		**18**		**30**		**32**	**33**	**34**	**36**	**41**	**48**	**49**	**51**	**53**	**54**	**59**	**60**	**61**	**66**	**69**	**74**	**75**	**77**	**80**	**82**	**83**	**84**	**85**	**89**	**91**	**92**	
**B**. ***cox*****1 amino acid substitution**.																																											
UAF1	G		F		F		V		G		V		L		H		G	F	S	Q	L	H	S	T	S	D	L	I	I	Y	P	G	D	G	E	Y	C	I	V	K	D	G	
UAF2	.		.		.		.		.		.		.		.		.	.	.	.	.	.	.	.	.	.	.	.	.	.	.	.	.	.	G	.	.	.	.	S	.	.	
UAF3	.		I		S		A		.		.		S		.		.	.	.	.	.	.	.	.	.	.	S	.	.	.	.	.	.	.	G	.	.	.	.	S	.	.	
UAF4	.		.		.		.		.		.		.		.		.	.	.	.	.	.	.	.	.	.	.	.	.	.	.	.	.	.	G	.	.	.	.	S	.	.	
UAF5	.		.		.		.		.		.		.		.		.	.	.	.	.	.	.	.	.	.	.	.	.	.	.	.	.	.	.	.	.	.	.	S	.	.	
UAF6	S		.		V		.		C		I		.		Y		W	V	G	E	F	N	W	S	W	N	.	T	V	C	S	C	C	R	G	H	Y	F	I	W	N	W	
UAF7	.		.		.		A		.		.		.		.		.	.	.	.	.	.	.	.	.	.	.	.	.	.	.	.	.	.	G	.	.	.	.	S	.	.	

Haplotype-1, -2, -3, -4, -5, -6, and -7 consisted of 10, 8, 7, 6, 4, 3, and 2 sequences, respectively.

The nucleotide diversity and neutrality indices for the entire *P. epiclitum* populations were calculated based on the sequences of the *cox*1 gene (Table 3). A low nucleotide (π) and high haplotype diversity (Hd) were observed for the *cox*1 gene. Tajima's *D* and Fu's Fs were negative and insignificant (*p* > 0.05).

**Table 3:** Diversity and neutrality indices for *Paramphistomum* populations from Pakistan.

***cox*1 (276 bp)**	
No. of isolates	40
No. of mutations	43
Parsimony informative sites	3
No. of haplotypes	7
Haplotype diversity (Hd)	0.756
Nucleotide diversity (π)	0.04693
Tajima's *D*	−1.50988
Fu's Fs	−1.120

**Figure 1:**
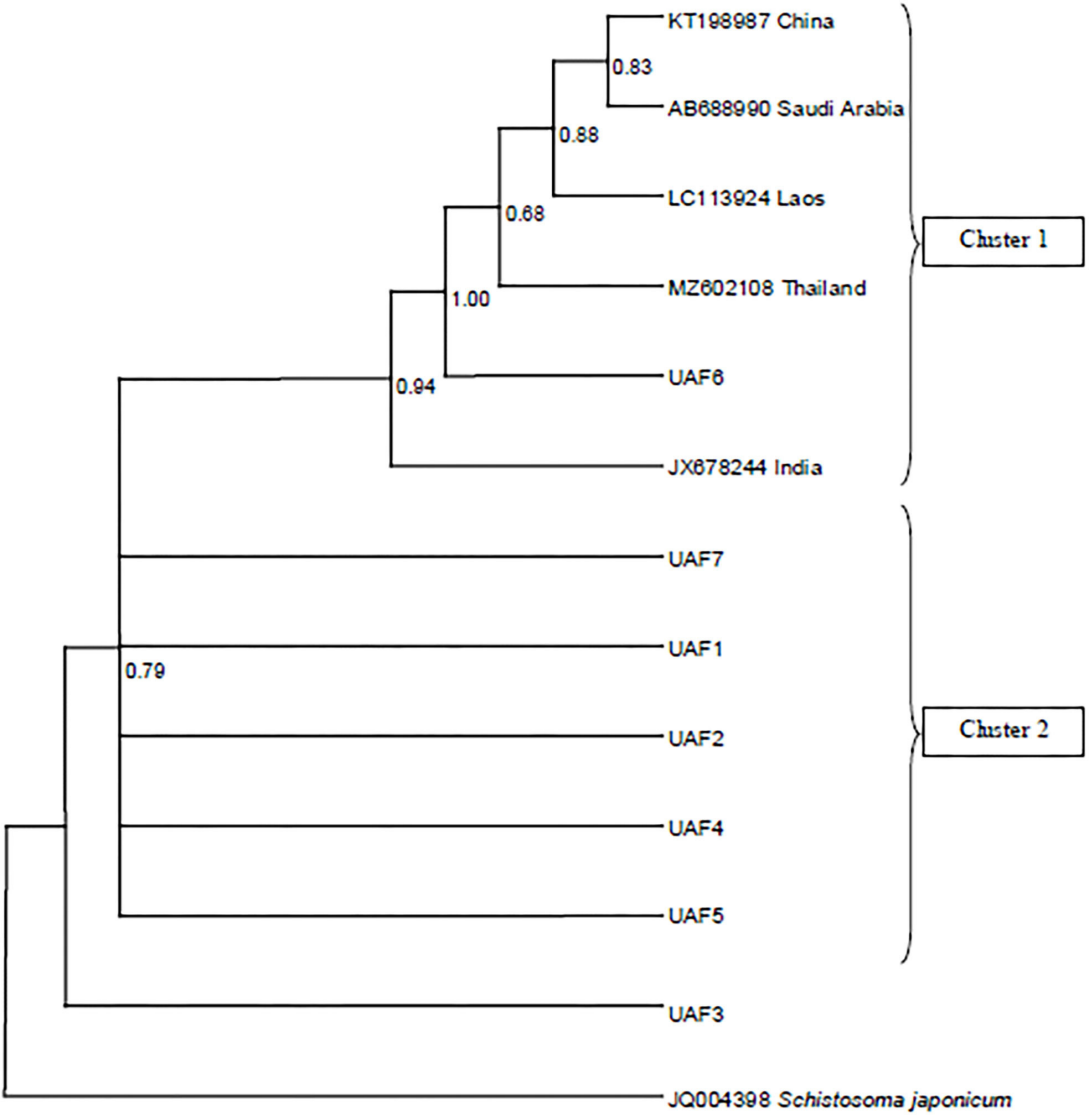
Bayesian phylogeny of Pakistani *Paramphistomum epiclitum* isolates inferred from the *cox*1 gene. *Schistosoma japonicum* is used as outgroup. Posterior probability values are depicted at the nodes.

The resulting sequences and those retrieved from GenBank were used to construct a phylogenic tree. The Bayesian phylogeny based on a dataset of the *cox*1 sequences placed most of the Pakistani *P. epiclitum* isolates in a distinct cluster (Figure 1). Only one sequence was found to be present within the cluster consisting of isolates from other countries [China (Accession No. KT198987), India (Accession No. JX678244), Laos (Accession No. LC113924), Saudi Arabia (Accession No. AB688990), and Thailand (Accession No. MZ602108)] of the globe.

Representative haplotype sequences from this study have been deposited in the GenBank database under the accession numbers ON899841–ON899847.

## Discussion

*Paramphistomum epiclitum* is one of the most important parasites affecting almost every livestock species. This parasite poses a huge economic loss to the livestock industry in terms of mortality and morbidity along with the losses in the production of wool, meat, and milk in infected animals which leads to decreased profitability for livestock holders (17).

Keeping in view the economic importance of *P. epiclitum* scattered efforts have been made to estimate the burden of *P. epiclitum* infection among different livestock species in different areas of the country. This study is also aimed at estimating the burden of *P. epiclitum* infection in small ruminants in District Faisalabad and adjoining localities along with the analysis of various parameters that might positively or negatively correlate or affect the presence of *Paramphistomum* infection in small ruminants.

The prevalence of *P. epiclitum* in sheep was calculated to be 28.6% while that in goats was found to be 24.50% and the rate of infection did not differ significantly among the species (*p* > 0.05). Similar results were also reported in a study in which reported a prevalence of 28.57% in sheep and 23.80% in goats (13). These findings were close to our study. A higher prevalence of 32.51 and 42% was reported in sheep and goats, respectively, from Andhra Pradesh, India (33). While (34) published a study that showed a lower prevalence of *P. epiclitum* in sheep and goats 7.4 and 4.95%, respectively, in Uttarakhand, India. Another study from Jammu showed that the prevalence *of P*. *epiclitum* is 30.9% in goats and 36.2% in sheep (35). Like all the previous studies, the prevalence of *P. epiclitum* calculated in our study was also slightly higher in sheep than that in goats but the difference was statistically insignificant. This difference in prevalence can be attributed to the difference in the grazing habits of both species as well as other factors such as management, nutrition, and deworming status of the herd which was not observed in this study.

Age group-wise prevalence in sheep showed the prevalence of 32.97, 30.51, 28.97, and 22.03% among different age groups of animals (<1 year, >4 years, 1–2 year, and 2–4 years). Calculation of age-wise prevalence in goats showed the prevalence of 27.13, 26.95, 23.05, and 22.10% among different ages groups of animals (<1 year, >4 years, 2–4 year, and 1–2 years) showing a non-significant (*p* > 0.05) difference in prevalence. A study conducted by Patel et al. (36) also found that animals aged <1 year were highly susceptible to *P. epiclitum* infection than the aged animals and reported a prevalence of 61.9% in animals of < 1 year age and 49.36% in aged animals. Similar findings of higher infection rates in young animals were also reported previously (37).

The study also calculated breed-wise prevalence among three breeds of sheep and prevalence was calculated to be 32.5, 27.78, and 25.61% among Buchi, Kajli, and Lohi breeds, respectively, and no significant (*p* > 0.05) difference was found in the rate of infection among different breeds of sheep. Similar results have also been reported in three other breeds of sheep named Balochi, Harnai, and Babrik. Prevalence was reported to be 21.5% in both Balochi and Harnai sheep while it was 17.75% in Babrik breed of sheep (37). Breed-wise prevalence in cross-bred, Teddy, and Beetal goats also differed non-significantly (*p* > 0.05) being 27.16, 22.14, and 21.47%, respectively.

Prevalence among female and male sheep was found to be 29.55 and 28.24%, respectively. While prevalence recorded in male and female goats were 25 and 23.77%, respectively. Results showed that the rate of infection was higher among female animals than that in male animals in sheep while it was higher in male animals in goats but no statistically significant (*p* > 0.05) difference in rates of infection was found among both sexes in sheep as well as goats. The study conducted by Iqbal et al. (38) on the prevalence of *Paramphistomum* in cattle represented a higher prevalence among male cattle than that in females being 9.67 and 5.79%, respectively. Prevalence of *Paramphistomum* was reported to be slightly higher in female sheep than that of male sheep and reported prevalence of 22.33 and 17.83%, respectively, in female and male sheep (39). Similar results were also reported by Kifleyohannes et al. (40), and reported the prevalence of 29.49% in females while 15.79% in male sheep. Contrasting results were reported by Tariq et al. (37) who reported a higher prevalence in male sheep than that in female sheep.

The prevalence of *P. epiclitum* was calculated to be significantly (*p* < 0.05) higher in animals that were dewormed than in those which did not receive any anthelmintic treatment. Prevalence in dewormed animals was 10.04% as compared to 47.68% in animals that were not dewormed. A higher gastrointestinal parasitic prevalence of 63% in animals that were not dewormed was reported as compared to 57% in those animals that were dewormed (41). Prevalence in grazing and stall-fed animals was 29.19 and 16.52%, respectively. Grazing animals had significantly (*p* < 0.05) higher rates of infection than stall-fed animals. While conducting a cross-sectional study to investigate fasciolosis also the study reported a higher prevalence in grazing animals than in animals that were not grazed (42).

Seasonal prevalence showed a significant (*p* < 0.05) relationship between season and prevalence of *P. epiclitum*. Prevalence was found to be highest during the Autumn months (36.95%; September to November) followed by Summer (30.97%; June to August), and Winter (17.12%; December to February). A similar trend has also been reported by Tariq et al. (43) but the prevalence was lower than that of our study. The study reported a prevalence of 8.33, 5.18, 2.98, and 1.17% during Autumn, Summer, Spring, and Winter respectively, in sheep while reported prevalence of 14.10, 9.02, 7.83, and 6.61% during Autumn, Summer, Winter, and Spring respectively, in cattle. The highest prevalence of 72.44% in the rainy season followed by 61.82% in Summer and 56.72% in Winter (44). While (45) reported the highest prevalence of 3.3% in Summer followed by 2.7% in Autumn, 2.3% in Winter, and 1.4% in Spring.

Infectious diseases such as parasitic infestations are important health problems in both animals and humans (46–50), which cause economic losses and severe illness (51–56). In this study, a molecular description of *P. epiclitum* isolates from sheep and goats was reported for the first time in Pakistan based on the mt *cox*1 gene as mtDNA remains an important marker in exploring intraspecific variation due to maternal inheritance, conserved structure, higher evolution rate, high genetic divergence, and absence of recombination (57–59). Previously, numerous reports on molecular characterization based on the ITS gene are available from the country but the *ITS* gene is a hypervariable region and thus does not a candidate gene to molecularly characterize and identify the species.

The phylogenetic analysis of the *cox*1 sequences from these isolates revealed a separate cluster suggesting the existence of unique *P*. *epiclitum* haplotypes in Pakistan. All the sequences obtained from GenBank were found to be in one cluster irrespective of the country of origin. To the best of our knowledge, this is the first report about the *cox*1 gene-based phylogeny of *P*. *epiclitum*. The scarcity of mt*cox*1 sequences from different geographical regions in the GenBank repository limited a wide-range comparison and interpretation.

Keeping in view the preliminary nature of this study and the logistic constraints encountered, only a segment of the *cox*1 gene was amplified. However, the previous reports suggest that to have a better insight into the population genetics structure of parasites, full-length gene amplification is preferred (60). Thus, investigations employing full-length gene amplification are highly warranted in the future.

## Conclusion

In this study, *P*. *epiclitum* was recovered from slaughtered sheep and goats in Faisalabad, Pakistan, and was characterized in what we believe is the first attempt to identify *P*. *epiclitum* in Pakistan based on mtDNA. The molecular analysis of the partial *cox*1 gene demonstrates a high degree of genetic variation with rare haplotypes among the Pakistani *P. epiclitum* population.

This study constitutes significant preliminary data for Pakistan as well as useful baseline information for future studies on the prevalence and population structure of *P. epiclitum* globally. The high prevalence of *P. epiclitum* demonstrates that more studies are indeed needed to further understand the prevalence and distribution of *P. epiclitum* in definitive and all potential intermediate hosts in addition to intraspecies variation and relationship with populations from other locations.

## Data availability statement

The datasets presented in this study can be found in online repositories. The names of the repository/repositories and accession number(s) can be found in the article/Supplementary material.

## Ethics statement

Ethical review and approval was not required for the study of animal participants in accordance with the local legislation and institutional requirements.

## Author contributions

MA, LL, H-BY, and W-ZJ conceptualized the study while methodology was designed by MA, W-ZJ, MS, and RA. Validation and formal analysis was carried out by MA, W-ZJ, LL, H-BY, MK, MI, and MS. Investigations were made by MA, FA, RA, AA, and WQ. The original draft was prepared by MA and FA. AA, HA, and NA revised the manuscript. Writing—review and editing of the manuscript was performed by W-ZJ, H-BY, and B-QF. Supervision, project administration, and funding acquisition were obtained by W-ZJ. All authors contributed to the article and approved the submitted version.

## Funding

We acknowledge funding received from the National Key Research and Development Program (2021YFE0191600), Cultivation of Achievements of State Key Laboratory of Veterinary Etiological Biology (SKLVEB2020CGPY01), and Central Public-Interest Scientific Institution Basal Research Fund (1610312020016).

## Conflict of interest

The authors declare that the research was conducted in the absence of any commercial or financial relationships that could be construed as a potential conflict of interest.

## Publisher's note

All claims expressed in this article are solely those of the authors and do not necessarily represent those of their affiliated organizations, or those of the publisher, the editors and the reviewers. Any product that may be evaluated in this article, or claim that may be made by its manufacturer, is not guaranteed or endorsed by the publisher.
