# Supplementary figures and images for: Prevalence, risk factors and first record of mitochondrial cox1 gene-based molecular characterization of Paramphistomum epiclitum from Pakistan
Source: Front Vet Sci. 2022 Nov 21;9:1018854. doi: 10.3389/fvets.2022.1018854 (PMC9719931; doi:10.3389/fvets.2022.1018854)

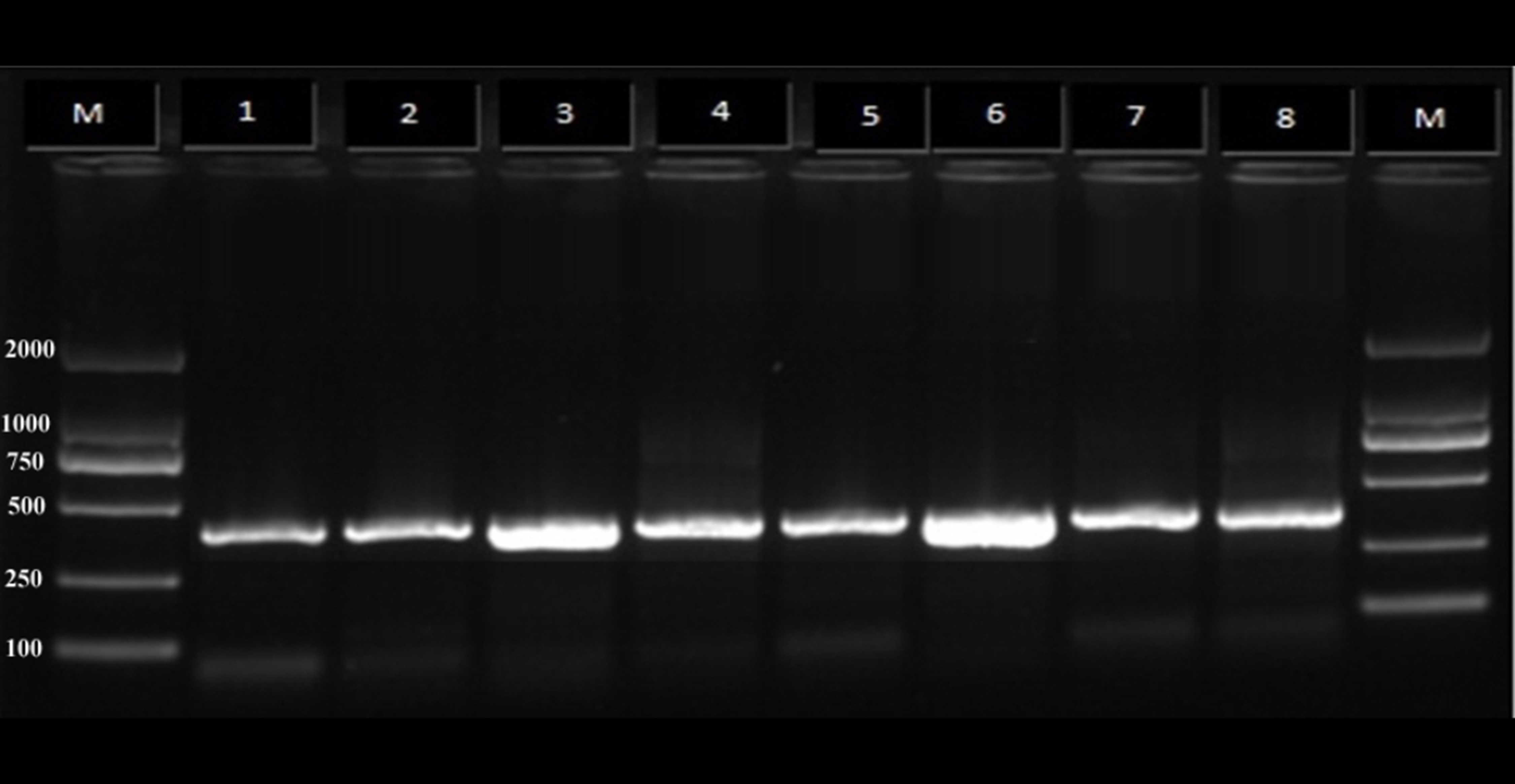

Supplement: Supplementary file 1 [file Image_1.JPEG]
